# Cliopatria - A geospatial database of world-wide political entities from 3400BCE to 2024CE

**DOI:** 10.1038/s41597-025-04516-9

**Published:** 2025-02-12

**Authors:** James S. Bennett, Erin Mutch, Andrew Tollefson, Ed Chalstrey, Majid Benam, Enrico Cioni, Jenny Reddish, Jakob Zsambok, Jill Levine, C. Justin Cook, Pieter Francois, Daniel Hoyer, Peter Turchin

**Affiliations:** 1 University of Washington, Seattle, WA USA; 2 Complexity Science Hub, Vienna, Austria; 3 University of California, Merced, CA USA; 4 The Alan Turing Institute, London, UK; 5 Murphy Institute, Tulane University, New Orleans, LA USA; 6 University of Oxford, Oxford, UK

**Keywords:** Geography, History

## Abstract

The scientific understanding of the complex dynamics of global history – from the rise and spread of states to their declines and falls, from their peaceful interactions with economic or diplomatic exchanges to violent confrontations – requires, at its core, a consistent and explicit encoding of historical political entities, their locations, extents and durations. Numerous attempts have been made to produce digital geographical compendia of polities with different time depths and resolutions. Most have been limited in scope and many of the more comprehensive geospatial datasets must either be licensed or are stored in proprietary formats, making access for scholarly analysis difficult. To address these issues we have developed Cliopatria, a comprehensive open-source geospatial dataset of worldwide states from 3400BCE to 2024CE. Presently it comprises over 1600 political entities sampled at varying timesteps and spatial scales. Here, we discuss its construction, its scope, and its current limitations.

## Background & Summary

With the advent of extensive on-line databases^1–3^ of curated historical information about productivity, population, trade, warfare, technology, etc., the scientific understanding of world-wide historical dynamics of states has made significant progress. For example, recent work has identified important causal regularities in the rise, spread, and fall of complex societies utilizing spatially-explicit models and exploring empirical evidence that incorporated geospatial and temporal information^4–7^. More traditional historical investigations have also engaged geospatial information, often concentrating on highly localized maps of particular cultures, or regional maps to highlight the spread of languages^8^ or growth in inter-regional exchange^9,10^. Similar efforts in economic history have utilized geo-spatial boundaries to explore the historical rise and spread of critical productive technologies and institutional packages argued as fundamental in the development of economic growth^11,12^. In lieu of good historical boundary data, however, these later efforts often use contemporary geo-spatial borders, which can mask important political developments in the past and can lead to measurement errors.

As these examples suggest, a wide range of studies across numerous disciplines depend upon and would benefit from a more comprehensive digital encoding of world-wide historical political geographies in time. Facilitating comparative analysis across social, spatial, and temporal bounds, including developing correspondences between different disparate geographical datasets, requires that the underlying geo-referenced data be represented as points, lines, and polygons in industry-standard digital encodings, such as shape or GeoJSON files.

Various attempts to construct such digital datasets have focused on a particular period or region of the world, digitizing maps at differing resolutions and sampling intervals^13–16^. However, these datasets are not comprehensive, even taken together^17^. Other efforts have sought to compile point-based data, which tend to be somewhat broader in scope but represent only one particular type of spatial information^18–21^. Yet other efforts^22^ catalog geo-referenced images of historical maps for visual inspection and summary but that limit automated computation of, for example, relative changes in political areas.

Several efforts, such as GeaCron^23^ or Running Reality^24^, have created more comprehensive world-wide digital representations of political entities over time. However, at present, their underlying data are not easily available to scholars. As of this writing they either require a license^23,25,26^, or they are encoded in a proprietary database scheme with limited options for exporting the data in other formats more amendable to computational analysis^19^. Further, to our knowledge, the references used in their construction are not available.

Here we describe Cliopatria, a comprehensive open-source geospatial digital dataset of worldwide polities — namely political units independent of higher authority which can range from city-states to empires, centralized or not^1^ — from 3400BCE to 2024CE. We describe the construction and contents of the initial version of the database, describe its validation, discuss how it compares to other digital databases, and note limitations and important considerations in its use. Subsequent versions of Cliopatria, which address these limitations (and any inaccuracies), will follow the established review procedures of the Seshat: Global Historical Databank project^1^.

## Methods

**Fig. 1:**
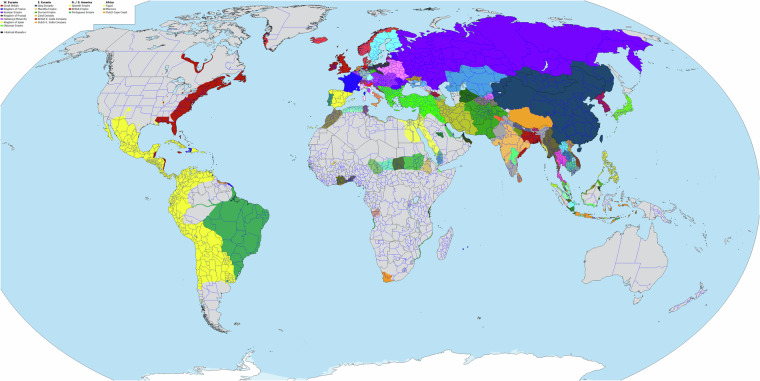
Example image for 1727CE. A partial legend of polities and their associated color is visible in the upper left corner. Modern interior land boundaries are shown in blue. Colored historical political regions are not aligned with those boundaries.

We initially created Cliopatria from a set of composite digital illustrations (map images) originally developed by one of us (AT) in 2014^27^. An extensive record was maintained of the documents used in the image set’s construction; these references, organized by modern state region, are listed in Table 1. The final image set consisted of 508 individual images, each associated with a specific year. An example map image, with its associated legend, is shown in Fig. 1. The complete map image set is available as part of the Cliopatria repository^28^.

**Table 1:** References used in the construction of the image set for Cliopatria.

Region	References
Canada	^36–40^
USA	^39,41–50^
Antilles	^51^
Mexico	^52–64^
Guatemala	^56,65^
Honduras	^66^
Nicaragua	^67,68^
Panama	^69^
Colombia	^70^
Venezuela	^71^
Peru	^72^
Bolivia	^73^
Paraguay	^74^
Chile	^74^
Argentina	^75,76^
Uruguay	^77,78^
Brazil	^79^
Europe & Periphery	^29–32,80^
Great Britain	^81–85^
France	^86–88^
Spain	^89–92^
Germany	^80,93,94^
Denmark and Sweden	^95,96^
Norway	^97,98^
Finland	^99^
Switzerland	^100^
Austria	^101,102^
Czech Republic	^103^
Hungary	^104,105^
Slovakia	^106^
Slovenia	^107,108^
Italy	^109–113^
Balkan Peninsula	^105,114–117^
Romania	^118^
Greece	^119,120^
Turkey	^121,122^
Ukraine	^123–125^
Poland	^126,127^
Russia	^128–133^
Georgia	^134^
Levant	^135^
Mesopotamia & Iran	^136,137^
Oman	^138^
Yemen	^139,140^
Egypt	^141^
Maghreb	^142^
Morocco	^143,144^
Sudan	^145,146^
Ethiopia	^147^
Somalia	^148^
Madagascar	^149^
Angola/Congo	^150,151^
Chad	^152^
West Africa	^153–155^
Turkmenistan	^156,157^
Ferghana	^158–163^
Afghanistan	^164^
Kashmir	^165^
Pakistan	^166–168^
India	^169–178^
Nepal	^170^
China	^171,179–193^
Mongolia	^159,186,194^
Korea	^195–197^
Japan	^198–207^
Vietnam	^187,208–211^
Cambodia	^212,213^
Laos	^214^
Thailand	^213–216^
Burma	^170,213,217–220^
Malaysia	^213,221–225^
Brunei	^226^
Philippines	^227–230^
Indonesia	^231–236^
Australia	^237^

To create these images the political boundaries for a given year found in the original source maps, typically in bound volumes, were redrawn by hand, as accurately as possible, onto a common, digital base map used by all images. Beginning with the Sumerian city states in 3400BCE, subsequent images were copied from the immediately preceding image and modified by hand to reflect documented incremental changes, additions, and deletions of polities in different geographical regions during a subsequent year as the literature suggested. Although various general world atlases^29–32^ suggested where political change occurred, more specialized regional sources (cited in Table 1) were consulted to confirm or resolve the detailed changes and to identify plausible and mutually consistent borders of abutting polities. Polities were included in an image when one or more written sources attested to its existence and provided an indication of its location and extent in particular years. As a consequence certain potential pre-historical polities (e.g., the ‘Xia Dynasty’ prior to 1600BCE) are not included. With rare exceptions (e.g., the Vatican, Singapore, various island states) polities occupy at least 5000 km^2^ and have a duration of at least 50 years.

**Fig. 2:**
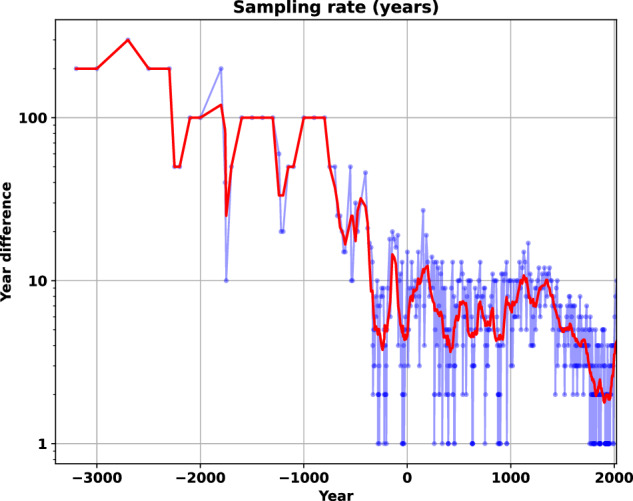
Original image sampling intervals. The time difference, in years, between images. Century moving average depicted in red. Note log scale.

The image set began in 3400BCE and ended in 2014CE but we extended the dataset to 2024CE. The images depict the intervening years irregularly depending on the information in the original sources and the number of events and major border changes that occurred in the year. Figure 2 shows the time difference (in years) between each image. Initially the images change information every few hundred years but the pace of change accelerates, sometimes changing on a yearly basis. Figure 2 thus provides a qualitative picture of periods of relatively stability of political boundaries compared with those with more frequent changes.

Most boundaries not associated with explicit treaties (e.g., the Peace of Westphalia in 1648) are necessarily approximate and subject to differing interpretations, even between text sources and digital repositories. In sparsely populated areas (e.g., nomadic confederations), boundaries were drawn conservatively in an attempt to reflect actual settlement patterns outlined in the sources. Further, political boundaries can change within a polity’s lifetime, typically as the result of documented occupation or treaties, and these are reflected in changes in images in the appropriate year (e.g., the widely-attested expansion of the Roman Republic under Julius Caesar into Hispania and Transalpine Gaul circa 50BCE is documented in images for this and preceding years^29^). The dataset does not currently encode possible border uncertainty and territorial disputes; this is discussed below.

To create the initial GIS dataset from these images, we developed Python code that converted the hand-colored regions on the images into polygons associated with the names in the accompanying legend. Then, with the generous assistance of researchers at the Seshat Databank project^1^, we extensively reviewed and hand-edited both the names and polygons and their associations to other datasets, notably Seshat, to form the Cliopatria database.

The original images have several unique advantages permitting the automated conversion to labelled polygons. First, each image uses an identical background image of the world. Land is marked in a grey; ocean and lakes in a light blue. The background map indicates coastal boundaries in black and modern internal land borders in blue and some currently disputed borders in red. The separately colored areas of historical polities, however, are not aligned with these modern interior boundaries. The map uses a (somewhat distorted but corrected) spherical Robinson projection (ESRI:53030), which permitted recovery of the approximate latitude and longitude of each image pixel. The dimensions of the image (2400 × 4800 pixels) provide a resolution of approximately 40 km^2^ at the equator.

Second, all text is restricted to the legend region in the upper left corner of the image and is not embedded in the world map itself. As a rule, the introduction of a new entity is announced in the legend, associated with a small rectangle of its color.

The uniformity of the background image permitted automated expansion of the entity color into adjacent inner border pixels. Initial polygons of uniform colors not associated with the background map were retrieved from the modified raster image. Certain small artifacts (of different colors) resulting from the original illustration process were identified and either associated with a related color (and hence entity) or were removed from the image.

Although the initial automated production of polygons from raster images yielded serviceable results, the distortions of the background image and the relatively coarse resolution provided by the images sometimes yielded polygons that are not always aligned with coastal and land region datasets. Further, to eliminate border artifacts from the raster-based images we automatically smoothed the resulting polygons and their shared borders to a 0.07° resolution. Subsequent releases will improve these alignment and resolution issues.

To associate an initial set of entity names with their accompanying colored polygon, we parsed the legend region using optical-character recognition (OCR) using the Tesseract library^33^, retrieving the text associated with each colored rectangle. The OCR process was largely successful but required detailed review and hand-editing to correct parsing artifacts (as when letters were distorted if they overlapped map boundaries) or when special characters were required. The legend area itself is constrained and did not always permit the listing of all the name or color changes in an image. Thus, the initial OCR legend data structure was subsequently edited by hand to add missing polities or disambiguate the names of polities in different regions.

The legend organized the world (and the polities) into four broad regions: Western Eurasia, Eastern Eurasia, Africa, and North/South America. While the location and extent of the latter two regions was clear, there was no clear boundary between Western and Eastern Eurasia. This led to some initial automated mis-assignment of names to polygons largely in Eastern Europe and in the Transcaucasian region. These were reviewed and corrected by hand.

Each polity was assigned one of 1194 unique colors, with images infrequently reusing the same color for different polities that existed at the same time in different regions of the world or at different times. Because colors were reused at different times and different regions of the world, it was possible for the initial automated process to mislabel polygons. For example, both the Chinese Jin and the Near-Eastern Neo-Assyrian polygons share the same color in the 750BCE image. However, the Neo-Assyrian polygons are partially in the Eastern Eurasia region, which led to automatically (mis)labeling these polygons as ‘Jin’ (or vice versa). To identify these issues we projected each Eurasian polity’s polygons individually (‘by name’) inspecting whether their extent over all their image years was consistent with the historical record; in the example above we would have found that the ‘Jin’ had an erroneous Near-Eastern presence, which was then corrected.

Certain polity names are reused in history at different times, e.g., ‘Jin’ refers to several Chinese states and dynasties over several millennia. Where possible we used known historical names utilized by experts in the relevant historical field to distinguish the different polities (e.g., ‘Western Jin’ from ‘Former Jin’).

In addition to polities the original images captured the occupation of territories by various leaders (e.g., Julius Caesar, Tokugawa Ieyasu), armies (e.g., the New Model Army, the Red Army), groups (e.g., the English Royalists and Parliamentarians) and the location of certain events (e.g., the Taiping Rebellion, the Sepoy Rebellion). We were often able to associate these entities with a particular polity (e.g., associating Harald Fairhair as the leader of the Old Kingdom of Norway from 866CE to 870CE). If this was the case we included the territory as part of the associated polity, otherwise it was not included in the current release of Cliopatria, pending further review.

**Fig. 3:**
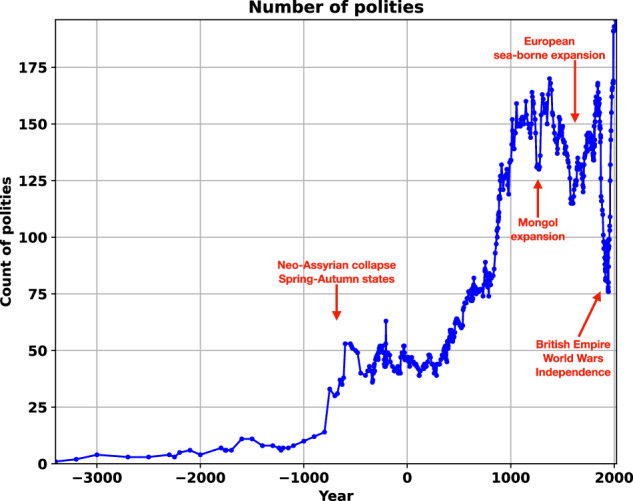
Number of entities depicted in each dataset year. Data reflects both the overall historical increase in number of polities but also a varying sampling choice about the scale of polities to be included. A substantial drop in polities typically reflects the expansion and occupation of states by a larger empire; a jump in polities reflects the creation or independence of states after the collapse of an empire. Notable examples of these dynamics are included (red lettering and arrows).

Figure 3 shows the number of polities recorded per image. Substantial changes in the number of polities over a short period of time reflects both sampling choices and the dynamics of empires absorbing and then releasing independent polities over their lifetimes.

For the initial release we have confined the database to the years recorded in the original image set and have largely respected the original choice of spatial and temporal resolution of political entities, which varies by region and the availability of original maps. Upon review, we improved the names of political entities and we sought to improve the representation of entities of certain areas, notably the Indian subcontinent. These improvements were based on expert historical knowledge provided by Seshat researchers. Subsequent releases will relax these constraints as additional suggestions, reviews, and investigations reflect, in accordance with standard Seshat review procedures^1^, modifications that increase historical accuracy and capture disputes and uncertainty.

## Data Records

The Cliopatria dataset is publicly available in a Zenodo repository^28^.

Cliopatria is distributed as a single data file, ‘cliopatria.geojson’. This file currently consists of approximately 15 K records structured as shown in Table 2. Data for each entity (e.g., ‘Roman Empire’) is contained in one or more rows, depending on how the associated data about the entity changes. Each row reports the **Name** of the entity, its polygons (**geometry**, projection EPSG:4326), and that geometry’s **Area** (in km^2^ using equal-area projection EPSG:6933).

**Table 2:** Example entity database entries.

Name	FromYear	ToYear	Area	Type	Wikipedia	SeshatID	MemberOf	geometry
a)								
Middle Kingdom of Egypt	−1800	−1701	395596.2	POLITY	Middle Kingdom of Egypt	eg_middle_k		POLYGON ((33.07 30.43,…
Hyksos	−1800	−1701	61952.4	POLITY	Hyksos			MULTIPOLYGON (((36.38 33.65,…
Middle Kingdom of Egypt	−1700	−1601	395596.2	POLITY	Middle Kingdom of Egypt	eg_middle_k		POLYGON ((33.07 30.43,…
Hyksos	−1700	−1601	91750.7	POLITY	Hyksos			POLYGON ((36.75 32.75,…
Fifteenth Dynasty of Egypt	−1600	−1501	34021.2	POLITY	Fifteenth Dynasty of Egypt	eg_thebes_hyksos		POLYGON ((30.64 27.54,…
Seventeenth Dynasty of Egypt	−1600	−1501	26544.0	POLITY	Seventeenth Dynasty of Egypt	eg_thebes_hyksos		POLYGON ((33.07 26.64,…
Sixteenth Dynasty of Egypt	−1600	−1501	22477.6	POLITY	Sixteenth Dynasty of Egypt	eg_thebes_hyksos		MULTIPOLYGON (((31.48 30.43,…
Hyksos	−1600	−1501	139092.8	POLITY	Hyksos			POLYGON ((36.75 32.75,…
New Kingdom of Egypt	−1500	−1401	634954.2	POLITY	New Kingdom of Egypt	eg_new_k_1		POLYGON ((34.35 31.30,…
Hyksos	−1500	−1401	66824.4	POLITY	Hyksos			POLYGON ((36.56 32.23,…
b)								
(British Empire)	1859	1859	10352664.6	POLITY	British Empire	gb_british_emp_2		MULTIPOLYGON (((−144.38 67.36,…
Kingdom of Great Britain	1856	1869	348055.9	POLITY	Great Britain	gb_british_emp_2	(British Empire)	MULTIPOLYGON (((−1.34 60.06,…
British Raj	1859	1867	4180749.1	POLITY	British Raj	gb_british_emp_2	(British Empire)	MULTIPOLYGON (((70.44 22.78,…
Trucial States	1856	1869	72118.9	POLITY	Trucial States	gb_british_emp_2	(British Empire)	POLYGON ((52.61 22.91,…
British Cape Colony	1856	1860	627919.5	POLITY	Cape Colony	gb_british_emp_2	(British Empire)	MULTIPOLYGON (((−16.67 13.07,…
British Colonial Empire	1859	1859	5124124.1	POLITY	British Empire	gb_british_emp_2	(British Empire)	MULTIPOLYGON (((−144.38 67.36,…
c)								
(Holy Roman Empire)	1305	1313	1047948.7	POLITY	Holy Roman Empire	de_empire_3		MULTIPOLYGON (((22.37 59.01,…
(Personal union of Kingdom of Bohemia with Kingdom of Poland)	1305	1313	305777.0	POLITY	Wenceslaus II of Bohemia	cz_bohemian_k_1; pl_piast_dyn_2		MULTIPOLYGON (((17.00 50.77,…
(Kingdom of Bohemia)	1305	1313	151557.3	POLITY	Kingdom of Bohemia	cz_bohemian_k_1		MULTIPOLYGON (((17.00 50.77,…
(Kingdom of Poland)	1305	1313	154219.6	POLITY	Kingdom of Poland	pl_piast_dyn_2		MULTIPOLYGON (((16.52 51.37,…
House of Luxembourg	1260	1362	7763.5	POLITY	House of Luxembourg	de_empire_3	(Holy Roman Empire)	MULTIPOLYGON (((4.56 50.37,…
County of Savoy	1260	1384	14693.8	POLITY	County of Savoy	de_empire_3	(Holy Roman Empire)	POLYGON ((6.66 45.10,…
Principality of Orange	1260	1458	233.8	POLITY	Principality of Orange	de_empire_3	(Holy Roman Empire)	POLYGON ((4.83 44.12,…
Patriarchate of Aquileia	1260	1313	9013.8	POLITY	Patriarchate of Aquileia	de_empire_3	(Holy Roman Empire)	MULTIPOLYGON (((13.73 46.34,…
Republic of Florence	1279	1401	9996.3	POLITY	Republic of Florence	it_florence_rep	(Holy Roman Empire)	POLYGON ((10.64 43.34,…
County of Brabant	1294	1401	13075.7	POLITY	Landgraviate of Brabant	de_empire_3	(Holy Roman Empire)	MULTIPOLYGON (((4.19 51.04,…
Swiss Confederation	1294	1325	2828.9	POLITY	Switzerland	de_empire_3	(Holy Roman Empire)	POLYGON ((8.89 47.26,…
House of Habsburg	1305	1313	60523.9	POLITY	House of Habsburg	de_empire_3	(Holy Roman Empire)	MULTIPOLYGON (((10.11 48.52,…
Duchy of Bavaria	1305	1325	35993.4	POLITY	Duchy of Bavaria	de_empire_3	(Holy Roman Empire)	MULTIPOLYGON (((7.02 49.51,…
Kingdom of Bohemia	1305	1313	151557.3	POLITY	Kingdom of Bohemia	cz_bohemian_k_1	(Holy Roman Empire); (Kingdom of Bohemia)	MULTIPOLYGON (((17.00 50.77,…
House of Ascania	1305	1332	28649.3	POLITY	House of Ascania	de_empire_3	(Holy Roman Empire)	POLYGON ((12.79 50.37,…
Holy Roman Empire Minor States	1305	1313	533920.4	POLITY	Holy Roman Empire	de_empire_3	(Holy Roman Empire)	MULTIPOLYGON (((11.95 51.04,…
Teutonic Order	1305	1313	111807.9	POLITY	Teutonic Order	pl_teutonic_order	(Holy Roman Empire)	MULTIPOLYGON (((22.37 59.01,…
County of Holland	1305	1351	11694.7	POLITY	County of Holland	de_empire_3	(Holy Roman Empire)	MULTIPOLYGON (((3.82 51.78,…
Margraviate of Brandenburg	1305	1313	41213.1	POLITY	Margraviate of Brandenburg	de_empire_3	(Holy Roman Empire)	POLYGON ((14.70 52.45,…
House of Wittelsbach	1305	1325	17716.5	POLITY	House of Wittelsbach	de_empire_3	(Holy Roman Empire)	POLYGON ((13.35 48.85,…
Duchy of Jawor	1305	1313	3556.1	POLITY	Duchy of Jawor	pl_piast_dyn_2	(Kingdom of Poland)	POLYGON ((16.50 50.97,…
Duchy of Legnica	1305	1313	4876.8	POLITY	Duchy of Legnica	pl_piast_dyn_2	(Kingdom of Poland)	POLYGON ((16.65 50.44,…
Duchy of Głogów	1305	1313	12372.0	POLITY	Duchy of Głogów	pl_piast_dyn_2	(Kingdom of Poland)	POLYGON ((16.52 51.37,…
Kingdom of Poland	1305	1313	133414.5	POLITY	Kingdom of Poland	pl_piast_dyn_2	(Kingdom of Poland)	MULTIPOLYGON (((17.75 53.73,…

Each row describes a polity over a range of years, its associated Wikipedia page, Seshat ID if any, and which composite polity the entity is a member of, if any. Area in km^2^. Geometry polygons are abbreviated. Components column is elided. (a) A subset of the sequence for ancient Egypt reflecting the invasion of the Hyksos and the collapse of the Middle Kingdom into the Second Intermediate Period, marked by several dynasties before the consolidation into the New Kingdom. (b) The major political constituents of the British Empire composite entity in the year 1859CE. (c) The entities associated with the Holy Roman Empire, the Kingdom of Poland, the Kingdom of Bohemia and the Personal Union between them under Wenceslaus II in 1305CE.

Each row indicates a range of years between **FromYear** to **ToYear** to which the associated row data applies. Years are recorded as integers, negative for BCE, positive for CE. Data, including polygons, for any entity for any year (not just original image years) between 3400BCE and 2024CE can be obtained finding the row (if any) containing the **Name** of the entity where the year of interest is between the row’s **FromYear** and **ToYear**, inclusive.

Each row also records an associated **Wikipedia** page (phrase) describing the entity in those years; the latter URL can be composed by embedding the phrase in “http://en.wikipedia.org/<phrase>”. For certain polities in particular years, an associated Seshat polity id (**SeshatID**) may be provided; access to the structured data about that polity can be found via the URL “http://seshat-db.org/core/polity/<polity_id>”.

In addition to associating an entity with a Seshat polity, some polities were parts of a larger political entity (e.g., the British Raj in India from 1859CE to 1947CE was part of the British Empire); thus polities can also have an associated (supra-) polity. Information about these associated entities are used to form *composite* polities, which are denoted in the database by enclosing their name in parentheses, e.g., “(Roman Empire)”. In addition, Seshat records some intra-polity relations, such as personal unions and political allegiances, which are also represented in the database as composites. Each entity, for a range of years, will list the composite entries it contributes to, if any, under the **MemberOf** column; each composite entity will list the member entities that contribute to it under **Components**. Polygons in **geometry** for composite entities duplicate those of its members. Examples of associated polity information and some resulting composites are shown in Table 2.

As noted, rows for an entity are added whenever any associated data for the entity changes; typically this happens because the spatial extent of the entity changes over time. There are, however, occasions when a (typically small) polity (e.g., the County of Navarre) is temporarily incorporated into a larger polity (e.g., the Kingdom of France) only for that larger polity to then shrink or collapse and expose the original polity once again. Thus there may be multiple rows for an entity with substantial gaps between the years recorded.

## Technical Validation

We validated the database largely by visual inspection and comparison against both the original and additional map images. We reviewed the image start and stop years for different entities with the original sources and with other databases, notably Seshat^1^. We also prepared various statistical summaries of the Cliopatria dataset to compare against previous such computations.

**Fig. 4:**
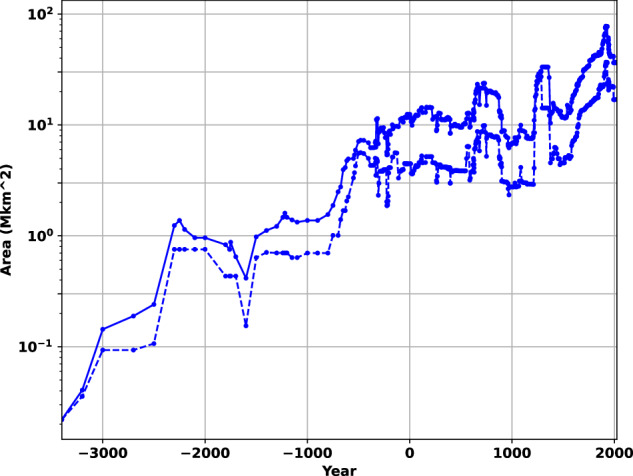
Size of polities versus time. The lower curve represents the area of the single largest polity. The upper curve is the sum of the areas of the three largest polities. Compare data to Taagepera^34^ Figs. 1 and 2. There is no essential difference. Maximum land area of the Earth is 133 M km^2^, excluding Antarctica; note log scale.

For example, in 1978 Taagepera^34^ prepared several extensive tables and figures based on his hand measurements of polity area from physical maps. We prepared equivalent tables and figures from our dataset; see Fig. 4, Tables 3–5. Our results are similar to Taagepera’s except that our database lists more steppe nomadic empires and those tend to replace his candidates for the largest empires during the Medieval period.

**Table 3:** Areas of the World’s Three Largest Empires before 0CE.

Year	Largest Empire		Second Largest Empire		Third Largest Empire	
3000BCE	Early Dynastic Period of Egypt	0.09	Sumerian City-States	0.04	Indus Valley Civilization	0.01
2950BCE	Early Dynastic Period of Egypt	0.09	Sumerian City-States	0.04	Indus Valley Civilization	0.01
2900BCE	Early Dynastic Period of Egypt	0.09	Sumerian City-States	0.04	Indus Valley Civilization	0.01
2850BCE	Early Dynastic Period of Egypt	0.09	Sumerian City-States	0.04	Indus Valley Civilization	0.01
2800BCE	Early Dynastic Period of Egypt	0.09	Sumerian City-States	0.04	Indus Valley Civilization	0.01
2750BCE	Early Dynastic Period of Egypt	0.09	Sumerian City-States	0.04	Indus Valley Civilization	0.01
2700BCE	Early Dynastic Period of Egypt	0.09	Sumerian City-States	0.08	Indus Valley Civilization	0.02
2650BCE	Sumerian City-States	0.08	Indus Valley Civilization	0.02	—	0.00
2600BCE	Sumerian City-States	0.08	Indus Valley Civilization	0.02	—	0.00
2550BCE	Sumerian City-States	0.08	Indus Valley Civilization	0.02	—	0.00
2500BCE	Sumerian City-States	0.11	Old Kingdom of Egypt	0.09	Indus Valley Civilization	0.04
2450BCE	Sumerian City-States	0.11	Old Kingdom of Egypt	0.09	Indus Valley Civilization	0.04
2400BCE	Sumerian City-States	0.11	Old Kingdom of Egypt	0.09	Indus Valley Civilization	0.04
2350BCE	Sumerian City-States	0.11	Old Kingdom of Egypt	0.09	Indus Valley Civilization	0.04
2300BCE	Indus Valley Civilization	0.75	Akkadian Empire	0.39	Old Kingdom of Egypt	0.09
2250BCE	Indus Valley Civilization	0.75	Akkadian Empire	0.53	Old Kingdom of Egypt	0.09
2200BCE	Indus Valley Civilization	0.75	Akkadian Empire	0.30	Elam	0.09
2150BCE	Indus Valley Civilization	0.75	Elam	0.09	Lower Egypt	0.07
2100BCE	Indus Valley Civilization	0.75	Gutian Dynasty	0.13	Lower Egypt	0.07
2050BCE	Indus Valley Civilization	0.75	Elam	0.02	Sumerian City-States	0.01
2000BCE	Indus Valley Civilization	0.75	Elam	0.11	Middle Kingdom of Egypt	0.09
1950BCE	Indus Valley Civilization	0.75	Elam	0.11	Middle Kingdom of Egypt	0.09
1900BCE	Indus Valley Civilization	0.75	Elam	0.11	Middle Kingdom of Egypt	0.09
1850BCE	Indus Valley Civilization	0.75	Elam	0.11	Middle Kingdom of Egypt	0.09
1800BCE	Middle Kingdom of Egypt	0.40	Indus Valley Civilization	0.25	Elam	0.14
1750BCE	Middle Kingdom of Egypt	0.40	Indus Valley Civilization	0.22	Babylonia	0.22
1700BCE	Middle Kingdom of Egypt	0.40	Babylonia	0.13	Hyksos	0.09
1650BCE	Babylonia	0.13	Hyksos	0.09	Elam	0.08
1600BCE	Shang Dynasty	0.15	Hyksos	0.14	Babylonia	0.13
1550BCE	Shang Dynasty	0.15	Hyksos	0.14	Babylonia	0.13
1500BCE	New Kingdom of Egypt	0.64	Mitanni	0.19	Shang Dynasty	0.15
1450BCE	New Kingdom of Egypt	0.64	Mitanni	0.19	Shang Dynasty	0.15
1400BCE	New Kingdom of Egypt	0.72	Babylonia	0.23	Mitanni	0.18
1350BCE	New Kingdom of Egypt	0.72	Babylonia	0.23	Mitanni	0.18
1300BCE	New Kingdom of Egypt	0.71	Hittites	0.33	Assyria	0.19
1250BCE	New Kingdom of Egypt	0.71	Hittites	0.33	Assyria	0.19
1200BCE	New Kingdom of Egypt	0.71	Assyria	0.46	Hittites	0.31
1150BCE	New Kingdom of Egypt	0.64	Assyria	0.54	Elam	0.22
1100BCE	New Kingdom of Egypt	0.64	Assyria	0.54	Shang Dynasty	0.15
1050BCE	New Kingdom of Egypt	0.64	Assyria	0.54	Babylonia	0.13
1000BCE	Zhou Dynasty	0.70	New Kingdom of Egypt	0.34	Kingdom of Kush	0.34
950BCE	Zhou Dynasty	0.70	New Kingdom of Egypt	0.34	Kingdom of Kush	0.34
900BCE	Zhou Dynasty	0.70	New Kingdom of Egypt	0.34	Kingdom of Kush	0.34
850BCE	Zhou Dynasty	0.70	Kingdom of Kush	0.34	Neo-Assyrian Empire	0.25
800BCE	Zhou Dynasty	0.70	Neo-Assyrian Empire	0.52	Kingdom of Kush	0.33
750BCE	Neo-Assyrian Empire	0.52	Chu	0.35	Kingdom of Kush	0.33
700BCE	Neo-Assyrian Empire	0.92	Kingdom of Kush	0.56	Chu Dynasty	0.35
650BCE	Assyrian Egypt	1.26	Chu Dynasty	0.35	Kingdom of Kush	0.34
600BCE	Median Kingdom	2.24	Neo-Babylonian Empire	0.71	Twenty-sixth Dynasty of Egypt	0.45
550BCE	Achaemenid Empire	3.33	Neo-Babylonian Empire	0.57	Twenty-sixth Dynasty of Egypt	0.47
500BCE	Achaemenid Empire	5.59	Chu Dynasty	0.35	Kingdom of Kush	0.33
450BCE	Achaemenid Empire	5.56	Chu	0.37	Kingdom of Kush	0.33
400BCE	Achaemenid Empire	5.03	Shaishunaga dynasty	1.56	Yuezhi	0.92
350BCE	Achaemenid Empire	4.36	Shaishunaga dynasty	1.56	Yuezhi	0.92
300BCE	Seleucid Empire	2.97	Maurya Empire	2.62	Yuezhi	0.92
250BCE	Maurya Empire	3.94	Seleucid Empire	2.76	Yuezhi	0.92
200BCE	Xiongnu	4.11	Seleucid Empire	2.27	Han Dynasty	2.04
150BCE	Xiongnu	5.12	Han Dynasty	2.04	Parthian Empire	1.86
100BCE	Xiongnu	3.33	Han Dynasty	3.15	Parthian Empire	2.20
50BCE	Han Dynasty	4.47	Parthian Empire	2.71	Xiongnu	2.40

Compare to Taagepera^34^ Table 2. Areas in M km^2^. Items depend on associating some polities with composite polities, e.g., British Empire.

**Table 4:** Areas of the World’s Three Largest Empires after 0CE.

Year	Largest Empire		Second Largest Empire		Third Largest Empire	
0CE	Han Dynasty	4.45	Roman Empire	3.63	Parthian Empire	2.71
50CE	Roman Empire	4.04	Han Dynasty	3.59	Parthian Empire	2.71
100CE	Han Dynasty	4.57	Roman Empire	4.42	Parthian Empire	2.71
150CE	Roman Empire	4.72	Han Dynasty	3.80	Xianbei	3.51
200CE	Xianbei	5.21	Roman Empire	4.80	Han Dynasty	3.42
250CE	Roman Empire	4.72	Sasanian Empire	2.70	Cao Wei	2.09
300CE	Western Jin	4.35	Roman Empire	4.32	Sasanian Empire	2.94
350CE	Roman Empire	4.33	Sasanian Empire	2.80	Western Jin	2.33
400CE	Sasanian Empire	2.99	Western Jin	2.43	Western Roman Empire	2.21
450CE	Rouran Khaganate	3.86	Sasanian Empire	3.14	Liu Song Dynasty	2.72
500CE	Rouran Khaganate	3.84	White Huns	3.44	Southern Qi	2.35
550CE	Sasanian Empire	3.20	Eastern Roman Empire	2.54	Liang Dynasty	1.93
600CE	Sui Dynasty	3.30	Western Göktürks	3.12	Sasanian Empire	3.06
650CE	Rashidun Caliphate	7.11	Tang Dynasty	6.55	Tibetan Empire	1.90
700CE	Umayyad Caliphate	8.62	Tang Dynasty	4.23	Türgesh	2.68
750CE	Umayyad Caliphate	5.23	Tang Dynasty	4.57	Uyghur Khaganate	2.94
800CE	Abbasid Caliphate	7.91	Tibetan Empire	3.90	Tang Dynasty	3.07
850CE	Abbasid Caliphate	7.89	Tang Dynasty	2.90	Tibetans	1.86
900CE	Tang Dynasty	3.10	Samanid Empire	2.54	Abbasid Caliphate	2.46
950CE	Samanid Empire	3.14	Five Dynasties and Ten Kingdoms	2.75	Liao Dynasty	1.92
1000CE	Northern Song	2.75	Ghaznavid Empire	2.30	Fatimid Caliphate	1.98
1050CE	Southern Song	2.75	Great Seljuk Empire	2.45	Liao Dynasty	1.92
1100CE	Great Seljuk Empire	3.09	Kimek-Kipchak confederation	3.04	Southern Song	2.78
1150CE	Kimek-Kipchak confederation	2.95	Kara-Khitans	2.61	Great Seljuk Empire	2.51
1200CE	Kimek-Kipchak confederation	2.95	Kara-Khitans	2.61	Ghurid Dynasty	2.39
1250CE	Mongol Empire	24.76	Southern Song	1.93	Mamluk Sultanate	1.53
1300CE	Yuan Dynasty	14.23	Golden Horde	4.97	Ilkhanate	3.97
1350CE	Yuan Dynasty	14.23	Golden Horde	4.94	Chagatai Khanate	2.45
1400CE	Ming Dynasty	4.92	Golden Horde	4.32	Timurid Empire	3.97
1450CE	Ming Dynasty	4.93	Khanate of Sibir	4.37	Four Oirats	3.97
1500CE	Mongol Khanate	4.56	Ming Dynasty	4.30	Khanate of Sibir	2.82
1550CE	Spanish Empire	4.09	Ming Dynasty	3.72	Ottoman Empire	3.34
1600CE	Spanish Empire	7.19	Tsardom of Russia	4.82	Ottoman Empire	4.37
1650CE	Tsardom of Russia	10.06	Spanish Empire	6.84	Qing Dynasty	4.08
1700CE	Tsardom of Russia	13.66	Qing Dynasty	8.86	Spanish Empire	7.58
1750CE	Russian Empire	15.08	Qing Dynasty	9.32	Spanish Empire	7.68
1800CE	Russian Empire	16.75	Qing Dynasty	12.51	Spanish Empire	11.73
1850CE	Russian Empire	21.11	Qing Dynasty	12.10	Empire of Brazil	7.79
1900CE	British Empire	27.35	Russian Empire	23.02	Qing Dynasty	10.37
1950CE	Union of Soviet Socialist Republics	22.43	French Africa	11.65	British Empire	10.68
2000CE	Russian Federation	16.96	Canada	10.08	United States of America	9.45

Compare to Taagepera^34^ Table 2. Areas in M km^2^. Items depend on associating some polities with composite polities, e.g., British Empire.

**Table 5:** The 20 Largest Empires of States That Ever Existed.

Rank	Name	Maximum size (M km^2^)	Peak date
1	British Empire	36.77	1920CE
2	Mongol Empire	27.99	1280CE
3	Union of Soviet Socialist Republics	23.63	1940CE
4	Russian Empire	23.02	1900CE
5	Republics of the Soviet Union	21.49	1920CE
6	Russian Federation	16.96	2000CE
7	Yuan Dynasty	14.23	1360CE
8	Tsardom of Russia	13.79	1710CE
9	Second French Empire	12.76	1920CE
10	Qing Dynasty	12.52	1810CE
11	Spanish Empire	11.74	1790CE
12	French Africa	11.65	1950CE
13	Canada	10.08	1960CE
14	Umayyad Caliphate	9.92	740CE
15	United States of America	9.62	1970CE
16	Abbasid Caliphate	8.19	780CE
17	Empire of Brazil	7.79	1850CE
18	Tang Dynasty	7.67	670CE
19	Rashidun Caliphate	7.11	650CE
20	Göktürk Khaganate	6.49	570CE

Compare to Taagepera^34^ Table 3. Entries depend on associating some polities with composite polities, e.g., British Empire.

**Fig. 5:**
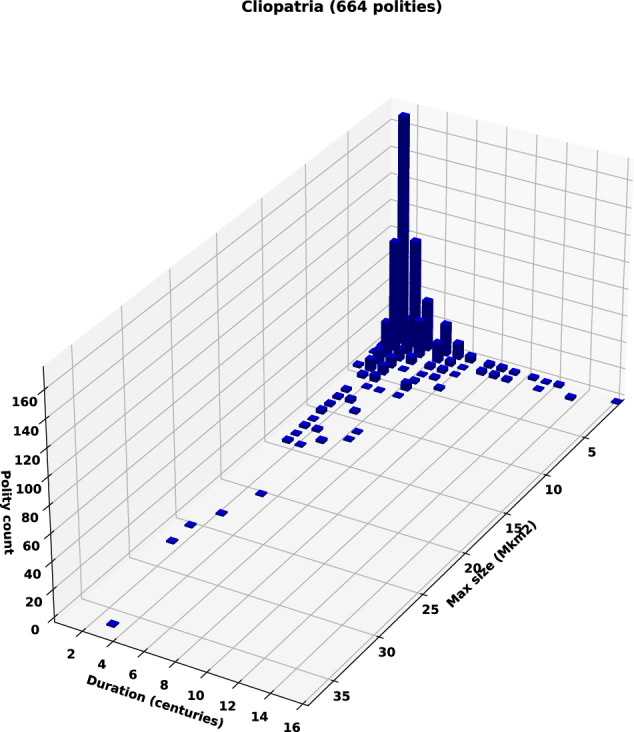
The distribution of the size and duration of polities from 3400BCE to 2024CE. Size in millions of km^2^; duration in centuries. Includes only polities that reach at least 100 K km^2^ and last for at least 50 years. Compare to Bennett^7^, their Fig. 5(A).

Bennett^7^ observed that the dramatic increase of polity size after 500BCE (also identified by Taagepera^34^) was not associated with an increase in polity duration. This pattern is confirmed by our more extensive database. Figure 5 shows the size and duration distribution of nearly 700 large-scale, long-duration polities over the 5400 years between 3400BCE and 2024CE. The very large polities exceeding 20 M km^2^ that arose after 1500CE (e.g., the British Empire) can clearly be seen but, again, the duration of most polities, including the largest, are just a few centuries.

**Fig. 6:**
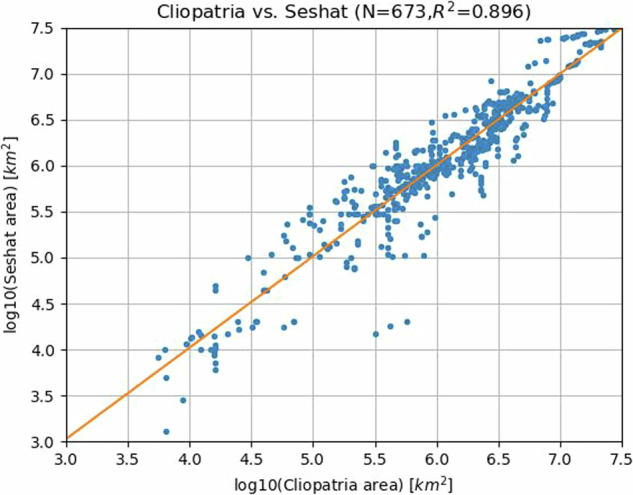
Comparison of unique paired Cliopatria and Seshat territory size estimates. Note log scales on both axes. Linear fit is shown in orange.

For those Cliopatria polities with associated Seshat database entries, we compared the area of Cliopatria’s entity polygons with Seshat polity territory data, if any, for the specific image years. The Seshat data comprise previously-collected, independent estimates of polities verified by historians and thus can serve as an indication of the variance in sizes present in Cliopatria. (Of course this comparison does not address the specific location and boundary extents for a polity; see below). The results of the comparison are shown in Fig. 6. The match is very good with a nearly 1:1 linear fit explaining nearly 90% of the variance, increasing our confidence in both datasets. We expect this value to increase as discrepancies are investigated. Indeed, performing this comparison identified several recent mis-coded entities in Seshat which had not yet been as thoroughly checked as older data and which are now corrected in Seshat. This demonstrates that Cliopatria, even in its early stages, is able to draw attention to discrepancies between databases leading to resolution. Further, research^35^ using the Seshat data has demonstrated that polity territory is a key proxy for social scale. The increased quantity and quality of the Cliopatria area data and its higher temporal resolution will permit more comprehensive investigations into the historical dynamics of social scale.

We also selectively compared Cliopatria’s entity polygons against several other available historical geospatial databases, both as a validation check of the Cliopatria records against previously-released resources as well as an indication of discrepancies between Cliopatria and different sources. Overall we find that while Cliopatria is at least comparable in data quality and coverage as other available databases, and often surpasses them in scope and comprehensiveness, there are gaps and disagreements between encodings in certain regions, especially in the existence and extent of Eurasian nomadic steppe empires.

**Fig. 7:**
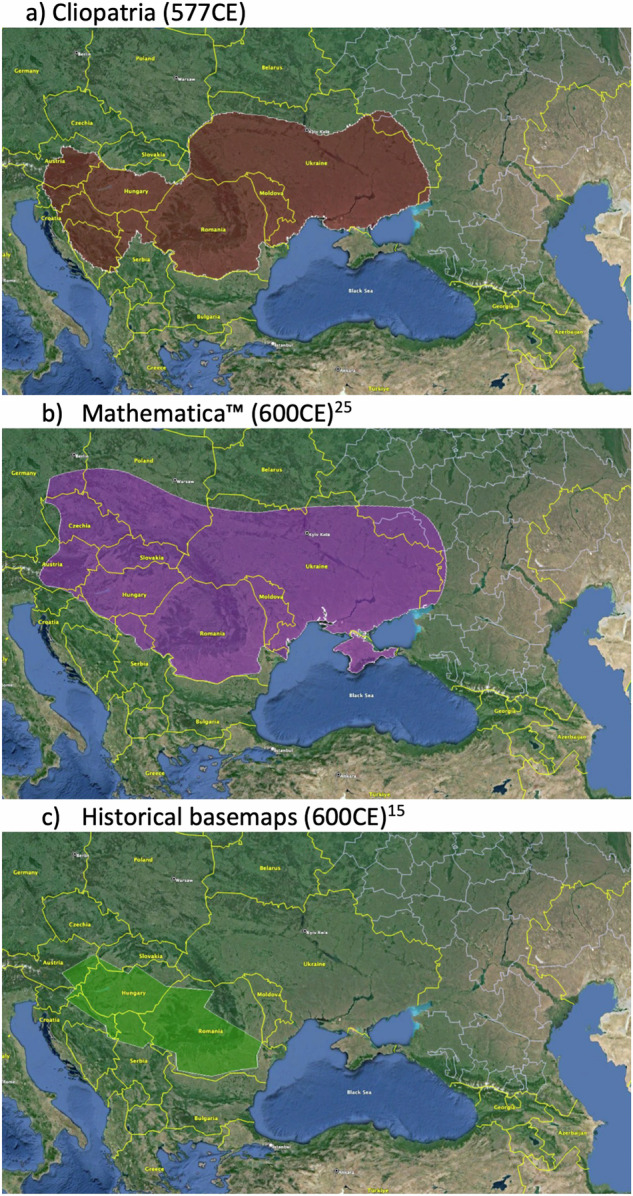
Comparison of the Avar Khaganate around 600CE. Putative extent of the Avars according to three different datasets. Background image from Google Earth showing modern state boundaries.

For example, Fig. 7 shows the polygons associated with the Avar Khaganate around 600CE for three different databases, including Cliopatria. While they all agree the Avars in this period occupied much of modern Romania and Hungary, their extent into modern Ukraine and Poland varies widely. This example shows that disagreements between databases can be substantial owing, no doubt, to the underlying procedures and sources referenced in their construction. The prevalence and magnitude of uncertain border locations of historical entities tends to increase into the past. Further, the apparent stability of borders of ancient polities over hundreds of years (as with Old, Middle, and New Kingdom Egypt) may reflect limited historical records rather than the actual stability of the state itself. This is typical of working with historical data, which is often fundamentally uncertain based on the simple paucity of records in addition to differing underlying concepts of border and control.

Many of the original source maps, which are themselves drawn by hand, employ different *cartographic* display techniques (e.g., stipple patterns, blurred edges, etc.) to suggest both the uncertain extent and location of some (but not all) borders. However, every known digital encoding of historical polity data, including Cliopatria, use industry-standard digital graphical primitives (raster encodings or polygons formed by latitude and longitude pairs) that are unable, by themselves, to capture this uncertainty which could then be used to inform display techniques or analytic computations. Further there is neither consistent discourse among historians about specific historical border uncertainties nor clear estimates of their location or rough magnitudes over time.

In spite of our attempts to reflect the most current historical knowledge, we acknowledge that Cliopatria’s representation of world history reflects only one version of the territories held by past polities. Thus, we warn users that currently unquantified uncertainty exists and they may need to account for it somehow in their analyses. We hope the availability and improvement of Cliopatria by the scholarly community will yield both improved borders based on documented input from historians and some broadly acceptable encodings of any residual uncertainty or disputes suitable for different computations, even if they are simply explicit alternative representations of the same polities. Indeed, one of the primary motivations of compiling the Cliopatria dataset and providing it as open-source material is to foster such productive, collaborative dialogue with other users and makers of historical geo-spatial information.

## Data Availability

Code to visualize the Cliopatria data in a Jupiter notebook is included in the Zenodo repository^28^.
